# Variability of physicians’ thresholds for neuroimaging in children with recurrent headache

**DOI:** 10.1186/1471-2431-14-162

**Published:** 2014-06-23

**Authors:** Carrie Daymont, Patrick J McDonald, Kristy Wittmeier, Martin H Reed, Michael Moffatt

**Affiliations:** 1 Department of Pediatrics and Child Health, The University of Manitoba, Winnipeg, MB, Canada; 2 The Manitoba Institute of Child Health, 655A-715 McDermot Avenue, Winnipeg, MB R3E 0Z2, Canada; 3 Section of Neurosurgery, The University of Manitoba, Winnipeg, MB, Canada; 4 The George and Fay Yee Centre for Healthcare Innovation, Winnipeg, MB, Canada; 5 Department of Radiology, The University of Manitoba, Winnipeg, MB, Canada; 6 Department of Community Health Sciences, The University of Manitoba, Winnipeg, MB, Canada

**Keywords:** Risk, Decision threshold, Clinical practice guideline, Medical decision-making, Headache

## Abstract

**Background:**

We sought to determine the extent to which physicians agree about the appropriate decision threshold for recommending magnetic resonance imaging in a clinical practice guideline for children with recurrent headache.

**Methods:**

We surveyed attending physicians in Canada practicing in community pediatrics, child neurology, pediatric radiology, and pediatric neurosurgery. For children in each of six risk categories, physicians were asked to determine whether they would recommend for or against routine magnetic resonance imaging of the brain in a clinical practice guideline for children with recurrent headache.

**Results:**

Completed surveys were returned by 114 physicians. The proportion recommending routine neuroimaging for each risk group was 100% (50% risk), 99% (10% risk), 93% (4% risk), 54% (1% risk), 25% (0.4% risk), 4% (0.01% risk). Community pediatricians, physicians in practice >15 years, and physicians who believed they ordered neuroimaging less often than peers were less likely to recommend neuroimaging for the 1% risk group (all p < 0.05).

**Conclusions:**

There is no consensus among pediatric specialists regarding the appropriate decision threshold for neuroimaging in a clinical practice guideline for children with recurrent headache. Because of the impact that individual threshold preferences may have on guidelines, these findings support the need for careful composition of guideline committees and consideration of the role of patient and family preferences. Our findings also support the need for transparency in guidelines regarding how evidence was translated into recommendations and how conflicts were resolved.

## Background

Variable recommendations for breast cancer screening among countries and organizations demonstrate the complexity of translating evidence into recommendations, even in very well-studied conditions [1-4]. Disagreement can arise over a variety of issues, including which studies provide sufficiently valid evidence to be included in analysis, the relative value of various outcomes, and the degree to which personal preferences of patients and families should be considered [5-9]. Another issue which may cause disagreement is the decision threshold: the level of risk above which testing or treatment should take place, and below which it is unnecessary [10-12].

Identification of the risk threshold for testing or treatment generally involves subjective judgment [13]. In some situations, decision analysis may help to identify an appropriate threshold. However, valid and reliable input required to obtain a valid and reliable result from decision analysis is unavailable for many conditions. Even when data are available, determining which outcomes should be considered in decision analysis, and how costs should be considered, involves some personal judgment. In practice, identification of a threshold may be entirely dependent on personal judgment, particularly for conditions with a relatively small evidence base regarding the natural history of disease and effects of treatment. This dependence on personal judgment may contribute to variability in the practice of individual physicians as well as variability in recommendations of clinical practice guidelines produced about the same topic.

Thresholds for action are an important part of clinical prediction rules. Clinical prediction rules are sometimes able to identify groups of patients with very high or low levels of risk for which the appropriate recommendation is clear. However, some groups of patients identified by a clinical prediction rule may have a degree of risk for which there is no consensus about the appropriate recommendation. For example, in a recent clinical prediction rule for identifying intracranial pathology in children with minor head trauma, approximately 30% of children in the study were found to have a combined risk of 0.9% for intracranial pathology [14]. The clinical prediction rule publication recommended making decisions based on individual factors for children in this intermediate-risk category.

In this study, we sought to explore the variability among physicians regarding decision thresholds. We performed a survey to identify the degree of consensus among physicians from relevant specialties about the appropriate threshold for neuroimaging in children with recurrent headache when forming a clinical practice guideline based on a clinical prediction rule. We also sought to explore physician characteristics that may be associated with recommendations for or against neuroimaging at a given risk level.

## Methods

### Survey design

No validated tools were identified to address our questions; therefore, a survey was developed by the research team. The survey was refined through two pilot surveys, administered to twelve physicians each, with three physicians contributing to both pilot surveys. The survey was administered via SurveyMonkey (Palo Alto, California) (Additional file 1).

We aimed to evaluate thresholds using a method that would best approximate decisions made during clinical practice guideline development. Participants were asked to respond as if they were part of a committee developing clinical practice guidelines for children with recurrent headaches based on a hypothetical, well-validated clinical prediction rule. Participants were advised that follow-up would be recommended regardless of the recommendation regarding neuroimaging. Participants were asked to indicate whether they believed magnetic resonance imaging should be recommended or not recommended for children in each of six risk categories: 50% (1/2), 10% (1/10), 4% (1/25), 1% (1/100), 0.4% (1/250) and 0.01% (1/10,000). The risk categories were chosen based on the pilot surveys, as well as a retrospective study of risk of pathology in children with headache and an associated cost-effectiveness analysis [15,16]. Participants were not provided with any corresponding clinical features for the hypothetical risk levels. An extremely high and an extremely low level of risk at which we expected no disagreement were included. Participants were also asked whether they would be willing to change their recommendation for the 1% risk group to achieve consensus if everyone else on the committee had chosen the opposite recommendation.

The final page of the survey included thirteen statements of beliefs about neuroimaging framed within the Theory of Planned Behavior [17-20]. Participants were asked to rate their level of agreement with the statements using a 7-point Likert scale. We initially identified 51 beliefs related to neuroimaging decisions based on literature and discussions with colleagues. In the interest of keeping the survey brief, we eliminated beliefs for which we anticipated a high degree of agreement, and included 13 belief statements in the final survey. The survey also included questions about advanced epidemiology training, participation in clinical practice guideline development, and the participants’ perception of his or her own neuroimaging ordering frequency compared to peers.

### Participants

The population of interest was physicians in Canada who are commonly involved in the care of children with recurrent headache or the pathology with which it may be associated. Attending physicians who were in active practice in one of the following four specialties were eligible for inclusion: community pediatricians, child neurologists, pediatric radiologists, and pediatric neurosurgeons.

Some community pediatricians in Canada practice primary care, although most see patients referred from family physicians. Two family physicians were included in the initial pilot, and both indicated that they would generally defer decisions about neuroimaging in children to pediatricians.

### Recruitment

Pediatric neurosurgeons were contacted through the email distribution list of the Canadian Pediatric Neurosurgery Study Group. Community pediatricians were contacted through the email distribution list for the Section of Community Pediatrics of the Canadian Pediatric Society. Pediatric radiologists were contacted through the Society for Pediatric Radiology. Child neurologist contact information was identified through publically available sources, and each was contacted individually. Review of the contact list by a Canadian child neurologist indicated that we identified the vast majority of attending Canadian child neurologists.

Each participant was contacted by email three times, at varying times and days of the week. The emails were sent 1–2 weeks apart. The tone and length of the emails also varied [21]. No monetary incentive was offered. No identifying personal information was collected except for an option to provide an email address in order to ask questions or request a copy of the results.

### Analysis

The primary outcome was the proportion of participants who would recommend neuroimaging for each risk category. The recommendation for the 1% risk category was used for further analysis because the highest level of disagreement was anticipated for this category. Fisher’s exact test was used to evaluate the association of eight physician characteristics and thirteen neuroimaging beliefs with the recommendation for the 1% risk category. Belief answers were converted to binary measures by combining all disagree and neutral answers in one category, and all agree answers in the other category. A p-value of 0.05 was used to determine statistical significance without a correction for multiple comparisons, as these analyses were exploratory and primarily for the purpose of hypothesis generation.

### Nonresponders and missing data

In order to evaluate for possible nonresponse bias, responses of physicians who responded to the first notice were compared with physicians who responded to the second or third notice [22]. The characteristics of physicians who did not respond to the primary question were also compared with the characteristics of those who responded fully.

### Ethics

Administration of the survey, and pilot surveys, was reviewed and approved by the Health Research Ethics Board at the University of Manitoba. The survey included a consent disclosure statement on the first electronic page (Additional file 1).

## Results

### Responses

The survey was administered between October 2011 and February 2012. The overall response rate for the survey was 35% (Table 1). The response rate varied by specialty. Pediatric neurosurgeons had a response rate of 84%. Pediatric neurosurgeons were relatively few in number and were contacted by one of the authors, who is also a colleague.

**Table 1:** Response and question completion rates, overall and by specialty

	**Overall**	**Pediatric neurosurgeons**	**Child neurologists**	**Pediatric radiologists**	**Community pediatricians**
**Total # initially contacted**	432	31	84	94	223
**# (%) responded**	152	26	33	25	68
	(35%)	(84%)	(39%)	(27%)	(30%)
**# respondents eligible**	132	24	30	18	60
**# (%) answered 1% threshold question**	114	21	26	11	56
	(86%)	(88%)	(87%)	(61%)	(93%)
**# (%) completed last question**	115	22	27	12	54
	(87%)	(92%)	(90%)	(67%)	(90%)

### Recommendations

No respondent had conflicting recommendations, defined as recommending neuroimaging for a group with a lower risk than a group for which they had recommended against neuroimaging.

For children with recurrent headache and a 1% risk of treatable pathology, 54% of surveyed physicians recommended routine neuroimaging and 46% recommended against routine neuroimaging (Table 2). Forty-five percent of the respondents indicated they would be willing to change their response for the 1% risk group in order to achieve consensus with the guideline committee. Respondents who recommended for neuroimaging in the 1% risk group were less likely to be willing to change their answer than those recommending against neuroimaging (33% v 58%, p = 0.008 using Fisher’s exact test).

**Table 2:** Recommendations for neuroimaging for each risk group, overall and by specialty

**Risk group**	**Percent and number recommending neuroimaging**				
	**Overall**	**Pediatric neurosurgeons**	**Child neurologists**	**Pediatric radiologists**	**Community pediatricians**
**50%**	100%	100%	100%	100%	100%
	116/116	23/23	26/26	11/11	56/56
**10%**	99%	96%	100%	100%	100%
	114/115	22/23	26/26	11/11	55/55
**4%**	93%	95%	96%	100%	89%
	107/115	21/22	25/26	11/11	50/56
1%*	54%	67%	65%	73%	39%
	61/114	14/21	17/26	8/11	22/56
**0.4%**	25%	23%	37%	36%	18%
	29/116	5/22	10/27	4/11	10/56
**0.01%**	4%	0%	8%	9%	4%
	5/113	0/22	2/24	1/11	2/56

*Denotes association of recommendation with specialty (p < 0.05 using Fisher’s exact test).

For the next-lowest risk category (0.4% risk) 25% of participants recommended routine neuroimaging. A small proportion of respondents (4%) recommended routine neuroimaging for patients in the lowest risk group (0.01% risk).

Most participants (93%) recommended routine neuroimaging for children with a 4% risk. All but one respondent recommended routine neuroimaging for children with a 10% risk of treatable pathology, and all recommended routine neuroimaging for children with a 50% risk of treatable pathology.

### Association of recommendation with physician characteristics and beliefs

Three of the eight tested characteristics were significantly (p < 0.05) associated with recommendation for the 1% risk group (Table 3). Those in practice more than 15 years were less likely than those in practice fewer than 15 years to recommend neuroimaging (41% v. 63% p = 0.023). Community pediatricians were less likely than subspecialists to recommend neuroimaging (39% v. 67%, p = 0.005). The response of community pediatricians did not vary by type of community practice (primary care versus consultant). Those physicians who believed that they ordered neuroimaging less often than their colleagues were less likely to recommend neuroimaging than those who believed they ordered neuroimaging at least as often as colleagues (35% v. 63%, p = 0.006).

**Table 3:** Association of physician characteristics with recommendation for 1% risk group

**Physician characteristic**	**Response**	**Total #**	**# (%)**	**# (%)**	**Fisher’s exact test**
			**Rec NI for 1%**	**Rec no NI for 1%**	
Specialty	Community pediatrician	56	22 (39%)	34 (61%)	
	Subspecialist	58	39 (67%)	19 (33%)	p = 0.005*
Gender	Male	62	33 (53%)	29 (47%)	
	Female	50	26 (52%)	24 (48%)	p = 1.000
Years in practice	15 or less	57	36 (63%)	21 (37%)	
	More than 15	54	22 (41%)	32 (59%)	p = 0.023*
Practice type	Academic	82	47 (57%)	35 (43%)	
	Community	32	14 (44%)	18 (56%)	p = 0.215
Practice location	Urban/suburban	99	55 (56%)	44 (44%)	
	Rural	15	6 (40%)	9 (60%)	p = 0.281
Advanced epidemiology training	Yes	17	11 (65%)	6 (35%)	
	No	96	49 (51%)	47 (49%)	p = 0.430
Participation in guideline production	Yes	69	37 (54%)	32 (46%)	
	No	45	24 (53%)	21 (47%)	p = 1.000
Self-assessment of imaging frequency	Less often than peers	43	15 (35%)	28 (65%)	
	More often or same	67	42 (63%)	25 (37%)	p = 0.006*

Two by two tables comparing characteristics with recommendation are presented along with p-values using Fisher’s exact test (<0.05 marked with *). Rec NI for 1% group = recommended routine neuroimaging for the 1% risk group; Rec no NI for 1% group = recommended against routine neuroimaging for the 1% risk group.

A high degree of variability was seen in the level of agreement for some of the belief statements, particularly those relating to patient and family comfort, anxiety, and preferences regarding neuroimaging and the degree to which those factors should be taken into account when making decisions about neuroimaging (Table 4). There were no significant associations between agreement with any of the belief statements and recommendation for the 1% risk group. There were also no significant associations between the evaluated physician characteristics or beliefs and willingness to change response in order to achieve consensus.

**Table 4:** Association of physician beliefs about neuroimaging with recommendation for 1% risk group

**Belief**	**Response**	**Total #**	**# (%)**	**# (%)**	**Fisher’s exact test**
			**Rec NI for 1%**	**Rec no NI for 1%**	
It would be possible to develop a clinical prediction rule that accurately determines risk for children with recurrent headaches.	Agree	97	54 (56%)	43 (44%)	
	Neutral/Disagree	17	7 (41%)	10 (59%)	p = 0.302
Neuroimaging is uncomfortable for many children.	Agree	66	32 (48%)	34 (52%)	
	Neutral/Disagree	48	29 (60%)	19 (40%)	p = 0.255
Patient comfort should be considered when making decisions about neuroimaging.	Agree	63	39 (62%)	24 (38%)	
	Neutral/Disagree	51	22 (43%)	29 (57%)	p = 0.059
Recommending neuroimaging is likely to cause anxiety for the patient or family.	Agree	70	36 (51%)	34 (49%)	
	Neutral/Disagree	44	25 (57%)	19 (43%)	p = 0.700
Recommending against neuroimaging is likely to cause anxiety for the patient or family.	Agree	73	42 (58%)	31 (42%)	
	Neutral/Disagree	41	19 (46%)	22 (54%)	p = 0.328
Patient and caregiver anxiety should be considered when making decisions about neuroimaging.	Agree	66	34 (52%)	32 (48%)	
	Neutral/Disagree	48	27 (56%)	21 (44%)	p = 0.705
The monetary cost to society should be considered when making decisions about neuroimaging.	Agree	82	43 (52%)	39 (48%)	
	Neutral/Disagree	32	18 (56%)	14 (44%)	p = 0.835
Caregivers of patients with recurrent headaches expect me to order neuroimaging.	Agree	62	37 (60%)	25 (40%)	
	Neutral/Disagree	52	24 (46%)	28 (54%)	p = 0.188
Patient or caregiver preferences should be considered when making decisions about neuroimaging.	Agree	59	35 (59%)	24 (41%)	
	Neutral/Disagree	55	26 (47%)	29 (53%)	p = 0.260
A delay in diagnosis leads to significant negative consequences for physicians.	Agree	95	52 (55%)	43 (45%)	
	Neutral/Disagree	19	9 (47%)	10 (53%)	p = 0.620
My colleagues believe it is important to avoid unnecessary neuroimaging.	Agree	96	48 (50%)	48 (50%)	
	Neutral/Disagree	18	5 (28%)	13 (72%)	p = 0.122
I am able to convince caregivers to agree with my point of view regarding whether their child should receive neuroimaging.	Agree	103	55 (53%)	48 (47%)	
	Neutral/Disagree	11	6 (55%)	5 (45%)	p = 1.000
I am able to determine which children require neuroimaging.	Agree	108	57 (53%)	51 (47%)	
	Neutral/Disagree	6	4 (67%)	2 (33%)	p = 0.684

Two by two tables comparing agreement with the belief with the recommendation are presented along with p-values using Fisher’s exact test (no p-values were <0.05). Rec NI for 1% group = recommended routine neuroimaging for the 1% risk group; Rec no NI for 1% group = recommended against routine neuroimaging for the 1% risk group.

We evaluated the 13 respondents with uncommon recommendations, including 5 who recommended for neuroimaging in the 0.01% risk group and 8 who recommended against neuroimaging in the 4% risk group (including 1 who also recommended against neuroimaging in the 10% risk group). Physicians with outlying responses of either type did not share any uncommon characteristics or beliefs. All 5 respondents who recommended for neuroimaging in the 0.01% risk group indicated that they did believe it was possible for a clinical prediction rule to accurately predict risk. Ten of 13 (77%) of these respondents with uncommon responses would not have agreed to change their answer for children with a 1% risk in order to achieve consensus.

### Late and incomplete responders

Physicians who responded to the first survey invitation were more likely to recommend neuroimaging for children with a 1% risk compared to physicians who responded to subsequent survey invitations (63% v 42%, Fisher’s exact p = 0.04). Physicians who responded to the first versus second or third survey invitations did not significantly differ regarding responses to any of the eight characteristics, agreement with belief statements, or willingness to change response in order to achieve consensus.

Eighteen of the 132 eligible respondents did not answer the primary question of interest regarding the recommendation for the 1% risk group. There were two physician characteristics associated with an increased likelihood of providing a response to the primary survey question regarding the recommendation for children with a 1% risk. Community pediatricians were more likely than specialists to answer the 1% question (93% v. 81%, p = 0.03), and those in community settings were more likely to answer the 1% risk question than those in academic settings (97% v 83%, p = 0.03).

### Comments

Several respondents mentioned in free text comments that it was difficult to answer some of the belief questions because they were dependent on circumstances. For example, a physician noted that if a child has a very high risk of pathology, parent preferences should not be considered but that in a patient with lower risk, parent preferences should be taken into account.

## Discussion

There is substantial disagreement among pediatric specialists regarding the appropriate recommendation for children with recurrent headache and a 0.4% or 1% of treatable pathology. Community pediatric practice, more than 15 years in practice, and self-perception of ordering neuroimaging less often than peers were significantly associated with a decreased likelihood to recommend routine neuroimaging for children with a 1% risk of treatable pathology. Respondents were mixed regarding their willingness to adjust their recommendations in order to achieve consensus with a guideline committee.

More research regarding the risks and benefits of neuroimaging in this population would potentially improve our ability to identify the best threshold for neuroimaging in children with recurrent headache, but some issues crucial for effective formal decision analysis will likely never be resolved. Most importantly, we will almost certainly never be able to quantify the impact of delayed diagnosis on the long-term outcomes of children with intracranial pathology who present with headache.

Our findings indicate that recommendations for children with intermediate degrees of risk may be strongly influenced by characteristics and beliefs of individual guideline committee members, particularly their beliefs about appropriate decision thresholds and the strengths of these beliefs. These findings provide support for recommendations from the Institute of Medicine and others for guidelines to include information about the methods for translating evidence into recommendations and also to describe how conflicts were resolved [5,23,24].

The findings also support recommendations that guideline development committee members should include a diverse representation of health care professionals in addition to other stakeholders [5,23-25]. Including physicians with variable durations of practice and ensuring representation from both academic and community practice may be factors to consider when evaluating the diversity of a committee. It may also be appropriate to consider identifying members with varied self-perceptions of practice style. Some organizations producing guidelines may even wish to consider more explicit evaluations or discussions regarding the decision threshold preferences of potential committee members. Organizations producing guidelines may want to insure that those with less common views are included on guideline committees. Others may feel that certain views about thresholds do not represent the values of the organization or that physicians with less common views would have a disproportionate impact on the recommendations.

Particularly when there is a lack of consensus among health care professionals regarding the appropriate recommendation, universal recommendations in a guideline may not be appropriate [23,26-28]. Our findings support the use of explicit discussions in guidelines regarding the role of patient and family preferences, as demonstrated in clinical practice guidelines recently produced by the American Academy of Pediatrics [29-32].

Our study had limitations, including a response rate of 35%. This low response rate is of particular interest because physicians who responded earlier were more likely to recommend neuroimaging for the 1% group than physicians who responded later, indicating possible nonresponse bias. However, our primary conclusion indicating disagreement among physicians regarding the appropriate recommendation for children with recurrent headache and a 1% risk of treatable pathology would very likely remain true even with a large degree of non-response bias. We identified no other differences in physician characteristics or beliefs between early and late responders, including no difference in the rate of participation in guideline production. It is possible that timing of response may be associated with some important characteristics that we did not evaluate, or for which we did not have the power to detect a difference.

Other limitations include that we only surveyed physicians, and we only surveyed those practicing in Canada. We did not do any repeat testing to determine the reliability of recommendations or agreement with beliefs. In future studies, we would ask respondents how often they would agree with a belief rather than how strongly they agree, as recommended by several respondents in free-text comments. We only presented the risk of treatable pathology, but many physicians may make decisions based on the risk of any pathology, even if it is not treatable [33]. We presented the risk in two formats simultaneously, and did not evaluate alternate methods of presenting the degree of risk. Physician responses to the survey may or may not reflect decisions they would make in real life. The fact that physicians’ self-assessment of their imaging frequency compared to their peers were significantly associated with their recommendations is one indication that disagreement regarding appropriate thresholds in the survey answers may reflect real-world behavior.

No association was present between recommendations and beliefs based on constructs from the Theory of Planned Behavior. This may have resulted from a lack of power to detect important differences, the way we asked about beliefs, or a true lack of association between these beliefs and decision thresholds in this context. In the interest of keeping the survey brief, we did not explore other factors that may affect decision thresholds, such as physician risk preference or risk tolerance, which have been shown to have variable associations with decision-making [34-40].

## Conclusion

There is no consensus among pediatricians and pediatric subspecialists in Canada regarding the appropriate neuroimaging recommendation for children with a 1% risk of intracranial pathology. More evidence regarding the risks of neuroimaging and the benefit of early identification of pathology may help to guide further recommendations, but more evidence is unlikely to resolve the variability completely. Further research into factors that affect physician decision thresholds and other factors that drive variability in guideline production and individual physician decision-making could lead to improvements in the guideline production process and provide information to researchers who hope to develop the evidence that supports guidelines. Organizations planning to produce clinical practice guidelines should anticipate differing opinions regarding the translation of evidence into guidelines due to variable decision thresholds and should ensure transparency regarding the methods used to select committee members, to determine the content and strength of recommendations, and to resolve conflicts.

## Competing interests

The authors declare that they have no competing interests.

## Authors’ contributions

CD contributed to the conception of the study and design of the survey, distributed the survey, analyzed and interpreted the data, drafted the manuscript, and approved the final manuscript as submitted. PM contributed to the design of the survey, recruited pediatric neurosurgeons, edited the manuscript for important content, and approved the final manuscript as submitted. KW interpreted the data, edited the manuscript for important content, and approved the final manuscript as submitted. MR contributed to the design of the survey, assisted with recruitment of pediatric radiologists, edited the manuscript for important content, and approved the final manuscript as submitted. MM contributed to the conception of the study and design of the survey, edited the manuscript for important content, and approved the final manuscript as submitted.

## Pre-publication history

The pre-publication history for this paper can be accessed here:

http://www.biomedcentral.com/1471-2431/14/162/prepub

## Supplementary Material

Additional file 1 Pediatric Neuroimaging Survey. Click here for file

## References

[B1] Screening for breast cancer U.S. Preventive Services Task Force recommendation statement Ann Intern Med 2009 151 10 716 726 W–236. doi: 10.1059/0003-4819-151-10-200911170-00008 19920272 10.7326/0003-4819-151-10-200911170-00008

[B2] Peres J Mammography screening: after the storm, calls for more personalized approaches J Natl Cancer Inst 2009 doi: 10.1093/jnci/djp496 10.1093/jnci/djp496 20023204

[B3] Smith RA Saslow D Sawyer KA Burke W Costanza M Evans WP Foster RS Hendrick E Eyre H Sener S American Cancer Society guidelines for breast cancer screening: update 2003 CA Cancer J Clin 2003 53 3 141 169 10.3322/canjclin.53.3.141 12809408

[B4] The Canadian Task Force on Preventive Health Care Recommendations on screening for breast cancer in average-risk women aged 40–74 years CMAJ 2011 183 17 1991 2001 doi: 10.1503/cmaj.110334 22106103 10.1503/cmaj.110334 PMC3225421

[B5] Brouwers MC Kho ME Browman GP Burgers JS Cluzeau F Feder G Fervers B Graham ID Grimshaw J Hanna SE Littlejohns P Makarski J Zitzelsberger L AGREE II: Advancing guideline development, reporting and evaluation in health care CMAJ 2010 doi: 10.1503/cmaj.090449 10.1503/cmaj.090449 PMC3001530 20603348

[B6] Barratt A Evidence based medicine and shared decision making: the challenge of getting both evidence and preferences into health care Patient Educ Couns 2008 73 3 407 412 doi: 10.1016/j.pec.2008.07.054 10.1016/j.pec.2008.07.054 18845414

[B7] Veatch RM Reasons physicians do not follow clinical practice guidelines JAMA 2000 283 13 1685 author reply 1686 10755485 10.1001/jama.283.13.1685

[B8] Woolf S Schünemann HJ Eccles MP Grimshaw JM Shekelle P Developing clinical practice guidelines: types of evidence and outcomes; values and economics, synthesis, grading, and presentation and deriving recommendations Implement Sci 2012 7 61 doi: 10.1186/1748-5908-7-61 10.1186/1748-5908-7-61 22762158 PMC3436711

[B9] Taylor JA Opel DJ Choriophobia: A 1-act play Pediatrics 2012 130 2 342 346 doi: 10.1542/peds.2012-0106 10.1542/peds.2012-0106 22778303 PMC8194452

[B10] Ben-Haim Y Zacksenhouse M Keren C Dacso CC Do we know how to set decision thresholds for diabetes? Med Hypotheses 2009 73 2 189 193 doi: 10.1016/j.mehy.2008.12.053 10.1016/j.mehy.2008.12.053 19349125

[B11] Pauker SG Kassirer JP The threshold approach to clinical decision making N Engl J Med 1980 302 20 1109 1117 10.1056/NEJM198005153022003 7366635

[B12] Graham ID Stiell IG Laupacis A O’Connor AM Wells GA Emergency physicians’ attitudes toward and use of clinical decision rules for radiography Acad Emerg Med 1998 5 2 134 140 10.1111/j.1553-2712.1998.tb02598.x 9492134

[B13] Jaeschke R Guyatt GH Sackett DL Users’ guides to the medical literature. III. How to use an article about a diagnostic test. B. What are the results and will they help me in caring for my patients? The Evidence-Based Medicine Working Group JAMA 1994 271 9 703 707 10.1001/jama.1994.03510330081039 8309035

[B14] Kuppermann N Holmes JF Dayan PS Hoyle JD Atabaki SM Holubkov R Nadel FM Monroe D Stanley RM Borgialli DA Badawy MK Schunk JE Quayle KS Mahajan P Lichenstein R Lillis KA Tunik MG Jacobs ES Callhan JM Gorelick MH Glass TF Lee LK Bachman MC Cooper A Powell EC Gerardi MJ Melville KA Muizelaar JP Wisner DH Zuspan SJ Identification of children at very low risk of clinically-important brain injuries after head trauma: a prospective cohort study Lancet 2009 374 9696 1160 1170 doi: 10.1016/S0140-6736(09)61558-0 10.1016/S0140-6736(09)61558-0 19758692

[B15] Medina LS Pinter JD Zurakowski D Davis RG Kuban K Barnes PD Children with headache: clinical predictors of surgical space-occupying lesions and the role of neuroimaging Radiology 1997 202 3 819 824 9051039 10.1148/radiology.202.3.9051039

[B16] Medina LS Kuntz KM Pomeroy S Children with headache suspected of having a brain tumor: a cost-effectiveness analysis of diagnostic strategies Pediatrics 2001 108 2 255 263 10.1542/peds.108.2.255 11483785

[B17] Walker AE Grimshaw JM Armstrong EM Salient beliefs and intentions to prescribe antibiotics for patients with a sore throat Br J Health Psychol 2001 6 4 347 360 doi: 10.1348/135910701169250 10.1348/135910701169250 12614509

[B18] Grimshaw JM Eccles MP Steen N Johnston M Pitts NB Glidewell L Maclennan G Thomas R Bonetti D Walker A Applying psychological theories to evidence-based clinical practice: identifying factors predictive of lumbar spine x-ray for low back pain in UK primary care practice Implement Sci 2011 6 1 55 doi: 10.1186/1748-5908-6-55 10.1186/1748-5908-6-55 21619689 PMC3125229

[B19] Millstein SG Utility of the theories of reasoned action and planned behavior for predicting physician behavior: a prospective analysis Health Psychol 1996 15 5 398 402 8891719 10.1037//0278-6133.15.5.398

[B20] Glanz K Rimer BK Viswanath K Health Behavior and Health Education: Theory, Research and Practice 2008 San Francisco: Jossey-Bass

[B21] Dillman DA Dillman DA Mail and internet surveys: the tailored design method 2000 New York: Wiley

[B22] Armstrong JS Overton TS Estimating nonresponse bias in mail surveys J Mark Res 1977 14 3 396 402 doi: 10.2307/3150783 10.2307/3150783

[B23] Clinical Practice Guidelines We Can Trust - Institute of Medicine Available at: http://www.iom.edu/Reports/2011/Clinical-Practice-Guidelines-We-Can-Trust.aspx. Accessed May 30, 2012

[B24] Eccles MP Grimshaw JM Shekelle P Schünemann HJ Woolf S Developing clinical practice guidelines: target audiences, identifying topics for guidelines, guideline group composition and functioning and conflicts of interest Implement Sci 2012 7 1 60 doi: 10.1186/1748-5908-7-60 10.1186/1748-5908-7-60 22762776 PMC3523009

[B25] Carpenter J Hutchings A Raine R Sanderson C An experimental study of the influence of individual participant characteristics on formal consensus development Int J Technol Assess Health Care 2007 23 1 108 115 doi: 10.1017/S0266462307051641 17234024 10.1017/S0266462307051641

[B26] Krahn M Naglie G The next step in guideline development: incorporating patient preferences JAMA 2008 300 4 436 438 doi: 10.1001/jama.300.4.436 18647988 10.1001/jama.300.4.436

[B27] Keirns CC Goold SD Patient-centered care and preference-sensitive decision making JAMA 2009 302 16 1805 1806 doi: 10.1001/jama.2009.1550 10.1001/jama.2009.1550 19861674

[B28] Opel DJ Taylor JA Phillipi CA Diekema DS The intersection of evidence and values in clinical guidelines: who decides what constitutes acceptable risk in the care of children? Hospital Pediatrics 2013 3 2 87 91 doi: 10.1542/hpeds.2012-0090 10.1542/hpeds.2012-0090 24340407

[B29] Kassirer JP Incorporating patients’ preferences into medical decisions N Engl J Med 1994 330 26 1895 1896 10.1056/NEJM199406303302611 8196734

[B30] Marcus CL Brooks LJ Draper KA Gozal D Halbower AC Jones J Schechter MS Sheldon SH Spruyt K Ward SD Lehmann C Shiffman RN Diagnosis and management of childhood obstructive sleep apnea syndrome Pediatrics 2012 130 3 576 584 doi: 10.1542/peds.2012-1671 10.1542/peds.2012-1671 22926173

[B31] Copeland KC Silverstein J Moore KR Prazar GE Raymer T Shiffman RN Springer SC Thaker VV Anderson M Spann SJ Flinn SK Management of newly diagnosed type 2 diabetes mellitus (T2DM) in children and adolescents Pediatrics 2013 131 2 364 382 doi: 10.1542/peds.2012-3494 10.1542/peds.2012-3494 23359574

[B32] Subcommittee on Attention-Deficit/Hyperactivity Disorder, Steering Committee on Quality Improvement and Management ADHD: clinical practice guideline for the diagnosis, evaluation, and treatment of attention-deficit/hyperactivity disorder in children and adolescents Pediatrics 2011 128 5 1007 1022 doi: 10.1542/peds.2011-2654 22003063 10.1542/peds.2011-2654 PMC4500647

[B33] Osmond MH Klassen TP Wells GA Correll R Jarvis A Joubert G Bailey B Chauvin-Kimoff L Pusic M McConnell D Nijssen-Jordan C Silver N Taylor B Stiell IG CATCH: a clinical decision rule for the use of computed tomography in children with minor head injury CMAJ 2010 182 4 341 348 doi: 10.1503/cmaj.091421 10.1503/cmaj.091421 20142371 PMC2831681

[B34] Nightingale SD Risk preference and laboratory use Med Decis Making 1987 7 3 168 172 10.1177/0272989X8700700307 3613916

[B35] Nightingale SD Risk preference and laboratory test selection J Gen Intern Med 1987 2 1 25 28 10.1007/BF02596246 3806268

[B36] Nightingale SD Risk preference and admitting rates of emergency room physicians Med Care 1988 26 1 84 87 10.1097/00005650-198801000-00009 3336247

[B37] Nightingale SD Grant M Risk preference and decision making in critical care situations Chest 1988 93 4 684 687 10.1378/chest.93.4.684 3349824

[B38] Andruchow JE Raja AS Prevedello LM Zane RD Khorasani R Variation in head computed tomography use for emergency department trauma patients and physician risk tolerance Arch Intern Med 2012 doi: 10.1001/archinternmed.2011.2243 10.1001/archinternmed.2011.2243 22450209

[B39] Allison JJ Kiefe CI Cook EF Gerrity MS Orav EJ Centor R The association of physician attitudes about uncertainty and risk taking with resource use in a Medicare HMO Med Decis Making 1998 18 3 320 329 10.1177/0272989X9801800310 9679997

[B40] Tubbs EP Elrod JA Flum DR Risk taking and tolerance of uncertainty: implications for surgeons J Surg Res 2006 131 1 1 6 doi: 10.1016/j.jss.2005.06.010 10.1016/j.jss.2005.06.010 16085105

