# Supplementary material for: Variability of physicians’ thresholds for neuroimaging in children with recurrent headache
Source: BMC Pediatr. 2014 Jun 23;14:162. doi: 10.1186/1471-2431-14-162 (PMC4079181; doi:10.1186/1471-2431-14-162)
Supplement: Additional file 1 — Pediatric Neuroimaging Survey. [file 1471-2431-14-162-S1.pdf]

Pediatric Neuroimaging Survey

You are being asked to participate in a research study. The purpose of the study is to understand how physicians make decisions about when neuroimaging should be performed. The survey is being conducted by Dr. Carrie Daymont, Dr. Patrick McDonald, Dr. Martin Reed, and Dr. Michael Moffatt and has been approved by the Research Ethics Board at the University of Manitoba.

Procedures: The study involves answering a series of questions online. We expect the survey to take between 5 and 10 minutes.

Risks and Benefits: We do not anticipate any risks from participating in this study. There will be no direct benefit to you from participating in this study. We hope the information learned from this study will be useful in planning future studies and for guideline development.

Confidentiality: We will not collect any personal identifiers and will collect a minimum of demographic information. We will not track your email or IP address.

Completion of the survey will be regarded as constituting implied consent to participate in the project. Your decision to take part in this study is completely voluntary.

Thank you for your time and consideration.

Are you an attending physician in active practice?

- ○ Yes
- ○ No

Would you describe your practice as any of the following? Please select all that apply.

- ☐ Family medicine with a practice that includes caring for children
- ☐ General pediatrics – outpatient consultant
- ☐ General pediatrics – inpatient
- ☐ General pediatrics – primary care
- ☐ Pediatric neurosurgery or neurosurgery with a practice that includes caring for children
- ☐ Child neurology
- ☐ Pediatric radiology
- ☐ None of the above

Did you participate in a pilot of this survey?

- ○ Yes
- ○ No

What is your gender?

- ◯ Male
- ◯ Female

How many years have you been in practice after completing all training?

In what type of area do you practice? Please select all that apply.

- ☐ Urban
- ☐ Suburban
- ☐ Rural
- ☐ Remote Northern
- ☐ Other (please specify)

In what setting(s) do you practice? Please select all that apply.

- ☐ Community hospital
- ☐ Community practice
- ☐ Private practice
- ☐ Academic hospital
- ☐ Academic practice network

Have you taken master's level coursework or obtained a master's degree or PhD in community health sciences, epidemiology, or public health?

- ◯ Yes
- ◯ No

Have you ever participated in the production of clinical practice guidelines? If so, were the guidelines for a local or broader audience? Please select all that apply.

- ☐ No guideline production participation
- ☐ Yes, guideline for my own institution
- ☐ Yes, guideline for a region
- ☐ Yes, guideline for a province
- ☐ Yes, guideline for Canada
- ☐ Yes, guideline for an international audience

Please complete the following statement.

Compared to my peers, I order neuroimaging:

- ○ Much less often
- ○ Less often
- ○ Slightly less often
- ○ As often
- ○ Slightly more often
- ○ More often
- ○ Much more often

For the remainder of the survey, please imagine that you are representing your peers on a committee to develop clinical practice guidelines for the care of children with recurrent headaches. The committee includes a variety of physicians. For this survey, you will be presented with data from a hypothetical clinical prediction rule and asked to make decisions based on that rule.

You and the other members of the committee are presented with a summary of the best available evidence regarding the risk of pathology in children with recurrent headaches. You learn that a validated clinical prediction rule has identified 6 groups of children with different risks of treatable pathology causing headache, such as brain tumors and hydrocephalus. If you are interested, more details about the rule are provided below the questions.

The committee is asked to use the information from this study to make recommendations to both primary care providers and specialists about when neuroimaging is appropriate, using the categories below. Ultimately the committee may choose to provide more graded levels of recommendation. However, at this stage you are asked to choose between “not recommended” and “recommended.”

Further Description of Recommendation Choices

Not recommended – The risk of treatable pathology is low enough that neuroimaging is not appropriate at this point. All patients should receive follow-up by the primary care provider and/or specialist. The need for neuroimaging should be re-evaluated if symptoms or findings on physical examination change.

Recommended – The risk of treatable pathology is high enough to warrant neuroimaging in these patients. All patients should receive follow-up by the primary care provider and/or specialist.

Please choose your recommendation for children with each degree of risk below.

Risk of treatable pathology 1 in 2 (50%)

◯ Not recommended ◯ Recommended

Risk of treatable pathology 1 in 10 (10%)

◯ Not recommended ◯ Recommended

Risk of treatable pathology 1 in 25 (4%)

◯ Not recommended ◯ Recommended

Risk of treatable pathology 1 in 100 (1%)

◯ Not recommended ◯ Recommended

Risk of treatable pathology 1 in 250 (0.4%)

◯ Not recommended ◯ Recommended

Risk of treatable pathology 1 in 10,000 (0.01%)

◯ Not recommended ◯ Recommended

More information about the clinical prediction rule for those who are interested

A large, multi-centre, prospective study has validated a clinical prediction rule to determine the risk of treatable pathology in children with recurrent headaches. The rule is applicable to well-appearing children and includes the following characteristics: age, duration of headaches, type of headache, elements of history (such as family history of migraine and vomiting) and physical examination (such as gait and focal neurologic abnormalities). It has been reviewed by multiple experts who agree that the study is of high quality and that the rule is valid in North American populations.

Imagine that after giving your answers, you find out that most of the other members of the committee felt differently than you did about the best recommendation for those children who had a 1% risk of treatable pathology causing headache.
The chair of the committee asks if you would be willing to endorse a guideline with the opposite recommendation. What would you say?

- ○ Yes
- ○ No

Last page -- you're almost done!!!

Please indicate your level agreement with the following statements about neuroimaging for children with recurrent headaches.

It would be possible to develop a clinical prediction rule that accurately determines risk for children with recurrent headaches.

○ Strongly disagree ○ Disagree ○ Slightly disagree ○ Neither agree nor disagree ○ Slightly agree ○ Agree ○ Strongly agree

---

Neuroimaging is uncomfortable for many children.

○ Strongly disagree ○ Disagree ○ Slightly disagree ○ Neither agree nor disagree ○ Slightly agree ○ Agree ○ Strongly agree

---

Patient comfort should be considered when making decisions about neuroimaging.

○ Strongly disagree ○ Disagree ○ Slightly disagree ○ Neither agree nor disagree ○ Slightly agree ○ Agree ○ Strongly agree

---

Recommending neuroimaging is likely to cause anxiety for the patient or family.

○ Strongly disagree ○ Disagree ○ Slightly disagree ○ Neither agree nor disagree ○ Slightly agree ○ Agree ○ Strongly agree

---

Recommending against neuroimaging is likely to cause anxiety for the patient or family.

○ Strongly disagree ○ Disagree ○ Slightly disagree ○ Neither agree nor disagree ○ Slightly agree ○ Agree ○ Strongly agree

---

Patient and caregiver anxiety should be considered when making decisions about neuroimaging.

○ Strongly disagree ○ Disagree ○ Slightly disagree ○ Neither agree nor disagree ○ Slightly agree ○ Agree ○ Strongly agree

The monetary cost to society should be considered when making decisions about neuroimaging.

○ Strongly disagree ○ Disagree ○ Slightly disagree ○ Neither agree nor disagree ○ Slightly agree ○ Agree ○ Strongly agree

Caregivers of patients with recurrent headaches expect me to order neuroimaging.

○ Strongly disagree ○ Disagree ○ Slightly disagree ○ Neither agree nor disagree ○ Slightly agree ○ Agree ○ Strongly agree

Patient or caregiver preferences should be considered when making decisions about neuroimaging.

○ Strongly disagree ○ Disagree ○ Slightly disagree ○ Neither agree nor disagree ○ Slightly agree ○ Agree ○ Strongly agree

A delay in diagnosis leads to significant negative consequences for physicians.

○ Strongly disagree ○ Disagree ○ Slightly disagree ○ Neither agree nor disagree ○ Slightly agree ○ Agree ○ Strongly agree

My colleagues believe it is important to avoid unnecessary neuroimaging.

○ Strongly disagree ○ Disagree ○ Slightly disagree ○ Neither agree nor disagree ○ Slightly agree ○ Agree ○ Strongly agree

I am able to convince caregivers to agree with my point of view regarding whether their child should receive neuroimaging.

○ Strongly disagree ○ Disagree ○ Slightly disagree ○ Neither agree nor disagree ○ Slightly agree ○ Agree ○ Strongly agree

I am able to determine which children require neuroimaging.

- ○ Strongly disagree
- ○ Disagree
- ○ Slightly disagree
- ○ Neither agree nor disagree
- ○ Slightly agree
- ○ Agree
- ○ Strongly agree

Thank you!!

We would welcome any additional comments about the survey, your answers or thought process.

If you would like a response or would like to receive the results of the study, you may include your email address or email Carrie Daymont at cdaymont@mich.ca.
